# Atividade Anti-Inflamatória *In Vivo* do D-Limoneno em um Modelo de Hipertensão Pulmonar Induzida por Monocrotalina em Ratos: Implicações à Função Cardíaca

**DOI:** 10.36660/abc.20240195

**Published:** 2024-11-22

**Authors:** Jorge Lucas Teixeira-Fonseca, Diego Jose Belato y Orts, Polyana Leal da Silva, Michael Ramon de Lima Conceição, Hernan Hermes, Carlos R. Prudencio, Danilo Roman-Campos

**Affiliations:** 1 Universidade Federal de São Paulo São Paulo SP Brasil Universidade Federal de São Paulo, São Paulo, SP – Brasil; 2 Instituto Adolfo Lutz São Paulo SP Brasil Instituto Adolfo Lutz, São Paulo, SP – Brasil; 3 Universidade São Paulo São Paulo SP Brasil Universidade São Paulo, São Paulo, SP – Brasil

**Keywords:** Hipertensão Pulmonar, Anti-Inflamatórios, Produtos Biológicos

## Abstract

**Fundamento:**

O D-Limoneno (D-L) é o principal monoterpeno monocíclico com propriedades anti-inflamatórias encontrado em plantas *citrus*. A Hipertensão pulmonar (HP) pode causar disfunção cardíaca direita e aumentar o risco de morte, em parte devido à resposta inflamatória no coração.

**Objetivo:**

Avaliar o possível efeito protetor do D-L sobre a função cardíaca em um modelo de HP induzida por Monocrotalina (MCT) (HP-MCT) em ratos.

**Métodos:**

Monitoramento eletrocardiográfico *in vivo*. A técnica de coloração tricromo de Masson foi empregada para verificar fibrose no coração. A função de contratilidade do tecido atrial foi estudada usando o banho de órgãos isolados. O PCR quantitativo em tempo real foi aplicado para quantificar inflamação no ventrículo direito.

**Resultados:**

O grupo HP-MCT mostrou remodelamento estrutural e elétrico do coração, com a presença de fibrose no tecido cardíaco e alterações eletrocardiográficas *in vivo*. O tratamento com D-L preveniu em parte o desenvolvimento de fibrose tecidual e o aumento na duração da onda P no grupo HP-MCT. As velocidades de contração e de relaxamento do átrio direito e do átrio esquerdo isolado foram aceleradas nos animais CTR e HP-MCT tratados com D-L. Finalmente, o D-L foi capaz de prevenir a expressão anormal das citocinas inflamatórias chave, interleucina 1-β, interleucina 6 e fator de necrose tumoral α, no ventrículo direito dos animais do modelo HP-MCT. D-L foi capaz de aumentar a produção da citocina anti-inflamatória interleucina-10.

**Conclusão:**

Nossos resultados mostraram que a administração *in vivo* de D-L preveniu em parte o remodelamento molecular, estrutural e funcional do coração no modelo HP-MCT com atenuação da reposta inflamatória no coração.

## Graphical abstract

**Figura Central:**
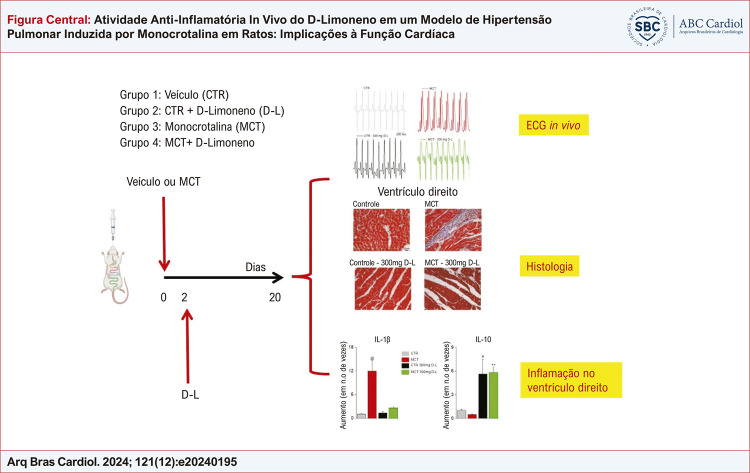
: Atividade Anti-Inflamatória In Vivo do D-Limoneno em um Modelo de Hipertensão Pulmonar Induzida por Monocrotalina em Ratos: Implicações à Função Cardíaca

A hipertensão pulmonar causa inflamação no coração e leva a um remodelamento funcional e estrutural do tecido. O componente natural D-limoneno administrado in vivo atenuou a resposta inflamatória no coração dos ratos no modelo de hipertensão pulmonar induzida por monocrotalina e melhorou a função e a estrutura do coração.

## Introdução

A Hipertensão pulmonar (HP) é uma doença crônica e multifatorial, caracterizada por um remodelamento progressivo do leito arterial pulmonar, resultando em disfunção da célula endotelial, proliferação anormal das células do músculo liso da artéria pulmonar, e presença de células inflamatórias na túnica adventícia.^1^ Essas alterações levam a um aumento da resistência vascular pulmonar e na pressão arterial pulmonar média.^2^ Em estágios mais avançados da HP, pode ocorrer uma sobrecarga de pressão sobre o ventrículo direito, contribuindo para hipertrofia e dilatação do coração direito e, eventualmente, a uma dificuldade de se manter o débito cardíaco.^3^

A HP induzida por Monocrotalina (MCT) (HP-MCT) em ratos é o principal modelo pré-clínico usado para se compreender a fisiopatologia da HP em humanos.^4^ A HP-MCT reproduz várias características do coração direito observadas em pacientes com comprometimento da função cardíaca secundário à HP, incluindo remodelamento elétrico e estrutural do miocárdio.^1,5,6^

Ao longo do curso da HP, lesões no miocárdio podem comprometer o sistema de condução elétrica, com o risco de desenvolvimento de arritmias, tanto em humanos como no modelo de HP-MCT em ratos.^7-10^ Estudos prévios sugeriram que vias apoptóticas, oxidativas e inflamatórias exercem um importante papel na patogênese da doença cardíaca atrial e ventricular observada na HP-MCT.^11-16^ Apesar de avanços significativos nas terapias farmacológicas^17^ e no manejo clínico das doenças, a probabilidade de sobrevivência dos pacientes com HP continua baixa.^18^ Portanto, é importante o desenvolvimento de novas terapias farmacológicas que visem reduzir o remodelamento eletromecânico do coração secundário à HP.

O D-Limoneno (4-isopropenil-1-metilciclo-hexeno), ou D-L, é um monoterpeno monocíclico encontrado predominantemente em óleos extraídos da casca de frutas cítricas, tais como limão, laranja e tangerina.^19^ Estudos mostraram que o D-L possui propriedades anti-inflamatórias e antioxidantes.^20-22^ Além disso, o D-L foi capaz de atenuar parte do remodelamento morfológico do coração no modelo de HP-MCT em ratos,^23^ embora os autores não tenham avaliado a função cardíaca nem o possível mecanismo cardioprotetor envolvido. Nesse sentido, especulamos que o D-L poderia reduzir a resposta inflamatória no coração e prevenir o desenvolvimento de remodelamento funcional e estrutural do tecido cardíaco após a administração de MCT em ratos.

## Métodos

### Animais

Todos os procedimentos de manipulação animal foram aprovados pela CEUA (Comissão de Ética no Uso de Animais) da Universidade Federal de São Paulo (#5438060923). Ratos pesando em torno de 100 g foram obtidos do CEDEME (Centro de Desenvolvimento de Modelos Experimentais para Medicina e Biologia) e alojados em instalações para cuidado animal em um ciclo claro/escuro de 12 horas, com alimento e água *ad libitum*.

### Delineamento experimental

Os ratos foram separados aleatoriamente em quatro grupos: 1) grupo controle (CTR): os ratos foram tratados com 1mL/Kg/dia de óleo de milho (veículo); 2) grupo MCT: os ratos receberem uma dose única de MCT 50mg/Kg (SIGMA Chemical Co. St. Louis, MO. EUA), via intraperitoneal (i.p.);^7-10^ 3) CTR+D-L: os ratos receberam 300 mg/Kg dia de D-L (SIGMA Chemical Co. St. Louis, MO, USA) diluído em óleo de milho; 4) MCT+D-L: após o tratamento com MCT (50mg/Kg, i.p.), os ratos receberam 300 mg/Kg/dia de D-L diluído em óleo de milho. Tratamentos com veículo e D-L foram administrados oralmente (gavagem) por 18-20 dias consecutivos, dois dias após a administração de MCT. Para evitar interferência com metabolismo do MCT, o tratamento com D-L foi iniciado dois dias após a injeção do MCT.^24^

### Experimentos nos átrios direitos e esquerdos isolados

Os átrios direitos e esquerdos foram cortados perpendicularmente e foram colocados em uma cuba para órgãos isolados contendo solução de Tyrode (em mM): 140 NaCl, 5,4 KCl, 1,8 CaCl_2_, 1,0 MgCl_2_, 0,33 NaH_2_PO_4_, 11 Dextrose e 5 HEPES que foi continuamente gaseificado com O_2_ 99,9% O_2_. As extremidades dos átrios foram suspensas horizontalmente por ganchos de aço inoxidável e equilibrados sob uma tensão de repouso de 0,5 gf (4,9 mN) por no mínimo 40 minutos. Somente o átrio esquerdo foi submetido a estímulo em campo elétrico (3 Hz, 100 V, 0,5 ms). A contração atrial foi normalizada usando o programa OriginLab.

### Exame histopatológico

Os corações foram removidos da cavidade torácica e lavados com solução salina tamponada com fosfato (PBS). Em seguida, foram fixados com paraformaldeído 4% por 24 horas, desidratado com etanol, lavados com xileno e embebidos em parafina. Os blocos de tecido fixados em parafina foram seccionados a uma espessura de 5 μm e desparafinizados por submersão em xileno, seguido de reidratação com álcool. As secções foram coradas com tricromo de Masson, seguindo as instruções do fabricante. Em seguida, as imagens foram adquiridas por um microscópio ótico (Leica DM4000B; Leica Microsystems). A área de fibrose do miocárdio foi quantificada usando o programa Image J (NIH, Bethesda, MD, USA).^8^

### Eletrocardiograma de superfície

Os ratos foram sedados com isoflurano 1,5-2,0% (Isoforine®, Cristália). Os exames eletrocardiográficos foram realizados em um aparelho ECG-PC versão 2,07 ^®^-TEB antes do início dos tratamentos e no último dia. A derivação II foi usada para calcular a duração da onda P, o complexo QRS, intervalos PR, intervalo QT e frequência cardíaca. Valore do intervalo QT foram corrigidos usando a fórmula QTc=QT/√RR. Todos os traçados eletrocardiográficos foram analisados *offline*. Para cada traço, as medidas foram tomadas em um minuto (média) de registro.

### Extração de RNA e análise de PCR Quantitativo em Tempo Real (RT-qPCR)

O RNA total foi extraído do tecido do ventrículo direito usando o reagente TRIzol LS (Life Technologies, Paisley, Reino Unido). O cDNA foi sintetizado usando *GoScript Reverse Transcription System* (Promega, Dübendorf, Suíça) de acordo com as instruções do fabricante. Quatro animais de cada grupo (CTR, MCT, CTR + D-L e MCT + D-L) foram usados para a análise da expressão celular de interleucina-1β (IL-1β), interleucina-6 (IL-6), interleucina-10 (IL-10), fator de necrose tumoral alfa (TNF-α), e o fator de crescimento transformador beta (TGF-β) por PCR quantitativo em Tempo Real (RT-qPCR) usando primers específicos^12^ e o kit GoTaq qPCR Master Mix (Promega). A expressão genética relativa foi normalizada para a expressão endógena de mRNA de β-actina por cada controle representativo.

RT-qPCR foi realizado em um sistema de PCR em tempo real ABI 7500 Fast real-time PCR (Applied Biosystems, Waltham, MA) com as seguinte condições: desnaturação inicial (5 min 95°C); desnaturação por 40 ciclos × 15 segundos à 95°C, anelamento/extensão do primer por 60 segundos a 60°C. A fluorescência foi registrada durante a etapa de anelamento/extensão em cada ciclo. Realizamos duas repetições técnicas para cada citoquina por grupo para avaliar a Quantificação Relativa (QR).^25^

A QR de um gene alvo em comparação a um gene de referência foi calculada de acordo com a equação $\mathrm{QR}\;=\;\frac{{{\mathrm{Eficiência}}_{\mathrm{alvo}}}^{∆\mathrm{Ct}\;\mathrm{alvo}\;(\mathrm{controle}\;-\;\mathrm{amostra}}}{{{\mathrm{Eficiência}}_{\mathrm{referência}}}^{∆\mathrm{Ct}\;\mathrm{referência}\;(\mathrm{controle}\;-\;\mathrm{amostra})}}$ .

### Análise estatística

Todos os dados foram apresentados como média ± erro padrão da média. A normalidade da variável dependente foi testada com o teste de Shapiro-Wilk test. Comparações entre os grupos foram realizadas usando a análise de variância de uma via ou de duas vias, e o teste de Tukey para comparação múltipla. O nível de significância para rejeitar a hipótese nula foi p<0,05. O número de animais foi representado por (N). Os dados foram analisados no Excel ® (Microsoft, EUA) e Origin 8,0^®^ (OriginLab, EUA).

## Resultados

### Administração *in vivo* de D-L não impede o comprometimento no ganho de peso induzido pelo MCT em ratos jovens

**Figura 1:**
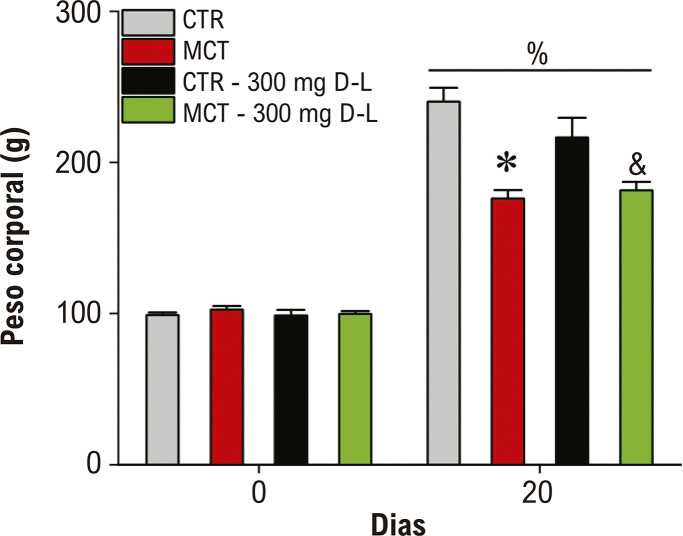
– Evolução do peso corporal ao longo do tempo. O peso corporal foi medido nos animais Controle (CTR), animais tratados com Monocrotalina (MCT), CTR + 300 mg/Kg/dia de D-Limoneno (D-L), e MCT + 300 mg/kg/dia de D-L no dia 0 e no dia 20. Dados são expressos em média ± erro padrão da média; p<0,05, ANOVA de duas vias (two way) para medidas repetidas seguido do pós-teste de Tukey, %comparando dia 0 e dia 20 com seu respectivo controle, *comparando CTR com MCT no dia 20, &comparando CTR 300 mg D-L com MCT 300 mg D-L no dia 20; N=4-6.

A administração de MCT causa alterações no ganho de peso em ratos.^8^ Assim, os animais foram pesados no início e no final do experimento. Um aumento no peso corporal foi observado em todos os grupos no dia 20 em comparação ao basal (Figura 1) (p<0,05). Contudo, 20 dias após a administração de MCT, o peso corporal dos animais dos grupos MCT e MCT+D-L estava reduzido em comparação aos grupos CTR e CTR+D-L, respectivamente.

### Remodelamento estrutural e elétrico cardíaco causado pelo MCT é parcialmente prevenido pela administração de D-L em ratos jovens

**Figura 2:**
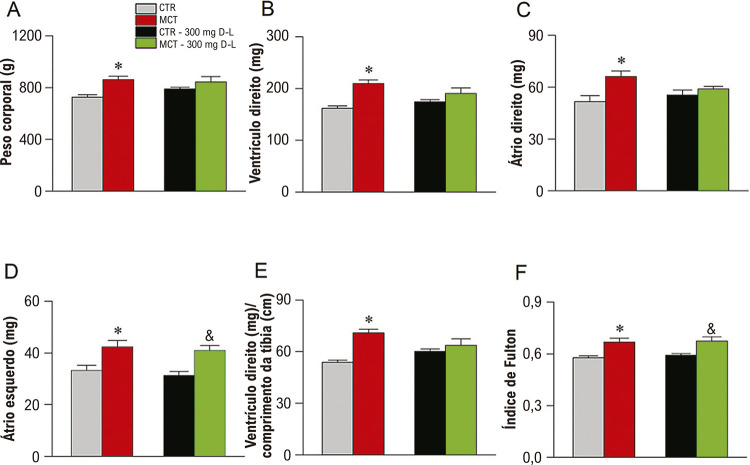
– Alterações morfológicas no coração. Grupos experimentais são Controle (CTR), Monocrotalina (MCT), CTR + 300 mg/Kg/dia de D-Limoneno (D-L), e animais tratados com MCT + 300 mg/Kg/dia de D-L. (A) Peso do coração. (B) Peso do Ventrículo Direito. (C) Peso do átrio direito. (D) Peso do átrio esquerdo. (E) Peso do ventrículo direito normalizado pelo comprimento da tíbia. (F) Índice de Fulton. Dados expressos em média ± erro padrão da média p<0,05, ANOVA de uma via seguido do pós-teste de Tukey *comparando CTR a MCT e & comparando CTR+D-L a MCT + D-L; N=4-5.

O remodelamento estrutural do coração é um marcador de HP-MCT em ratos.^7-10^ As medidas do peso do coração, do ventrículo direito, do átrio direito, do átrio esquerdo, da razão entre ventrículo direito e do comprimento da tíbia, e do índice de Fulton mostrou aumentos significativos no grupo MCT em comparação ao CTR (Figura 2A-F). Ainda, o peso médio do átrio esquerdo e o índice de Fulton foram mais altos nos animais MCT+D-L em comparação aos animais CTR+D-L (Figuras 2D e 2F).

**Figura 3:**
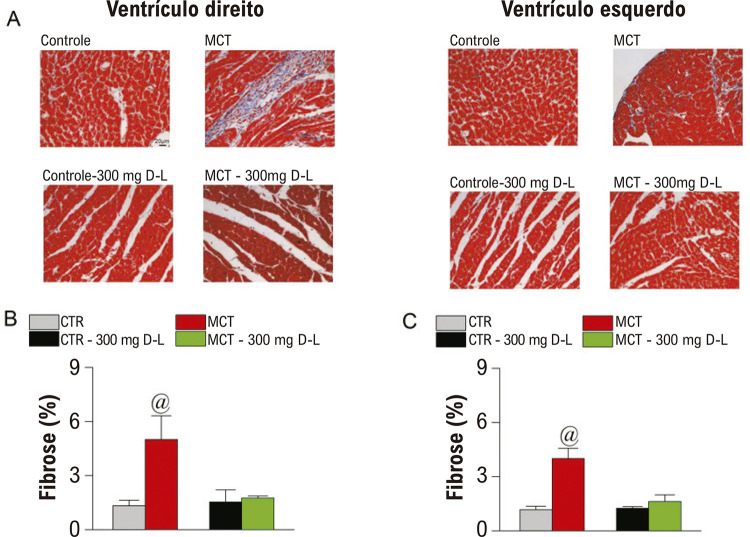
– D-Limoneno (D-L) atenua fibrose no coração. Grupos experimentais são Controle (CTR), Monocrotalina (MCT), CTR + 300 mg/Kg/dia de D-L, e animais tratados com MCT + 300 mg/Kg/dia de D-L. no dia 0 e no dia 20 (A) Seção representativa do ventrículo direito e do ventrículo esquerdo; (B) Fibrose no coração direito; (C) fibrose no ventrículo esquerdo. Dados são expressos em média ± erro padrão da média p<0,05, ANOVA de uma via seguido do teste de Tukey, @ comparando MCT a todos os outros grupos.

Já está bem documentado que um aumento na fibrose cardíaca é comumente observado nos animais tratados com MCT.^11-16^Assim, foram realizadas análises histológicas e os resultados para o ventrículo direito e esquerdo são ilustrados na Figura 3A. Os animais MCT apresentaram aumento na fibrose (Figuras 3B e 3C) em ambos os ventrículos, enquanto o tratamento com D-L atenuou a fibrose em ambos.

**Figura 4:**
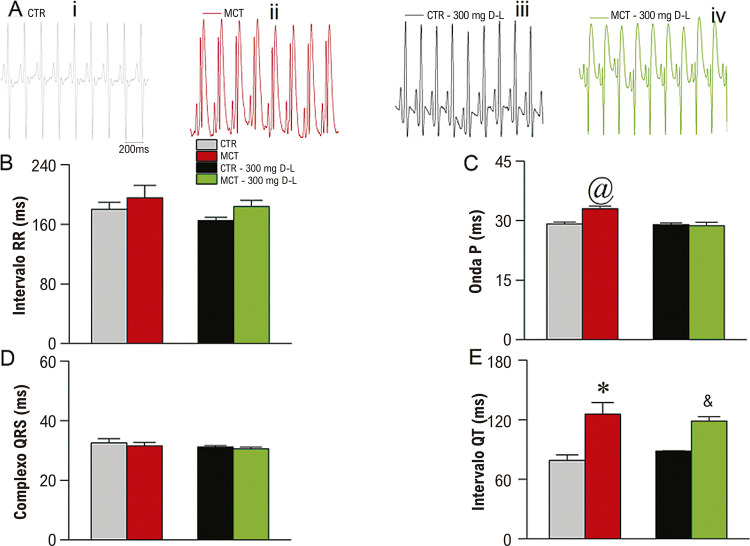
– Mudanças eletrocardiográficas. (A) Traços representativos do eletrocardiograma na derivação DII para Controle (CTR (i), Monocrotalina (MCT) (ii), CTR + 300 mg/Kg/dia de D-Limoneno (D-L) (iii), e animais tratados com MCT + 300 mg/kg/dia de D-L no dia 0 e no dia 20 (iv). (B) intervalo RR. (C) duração da onda P. (D) Complexo QRS. (E) intervalo QT; dados expressos em média ± erro padrão p<0,05, teste ANOVA de uma via seguido do teste de Tukey, @ comparando grupo MCT a todos os grupos, * comparando CTR a MCT e & comparando CTR+D-L a MCT+D-L. N=4-5.

Em seguida, exploramos o perfil eletrocardiográfico dos animais, que está resumido na Figura 4. Na (Figura 4A, i-iv) mostra traçados eletrocardiográficos representativos dos quatro grupos experimentais no dia 20. Como mostrado na (Figura 4B), não foram observadas mudanças na frequência cardíaca, medido como o intervalo RR, nem no complexo QRS (Figura 4D). A administração de MCT causou um aumento na duração da onda P (Figura 4C), e o tratamento *in vivo* com D-L preveniu o fenótipo (grupo MCT+D-L). Além disso, o intervalo Qt aumentou no grupo MCT em comparação ao grupo CTR (Figura 4D), e o tratamento *in vivo* com D-L não preveniu o alongamento do intervalo QT.

### D-L modula a função atrial direita e esquerda

**Figura 5:**
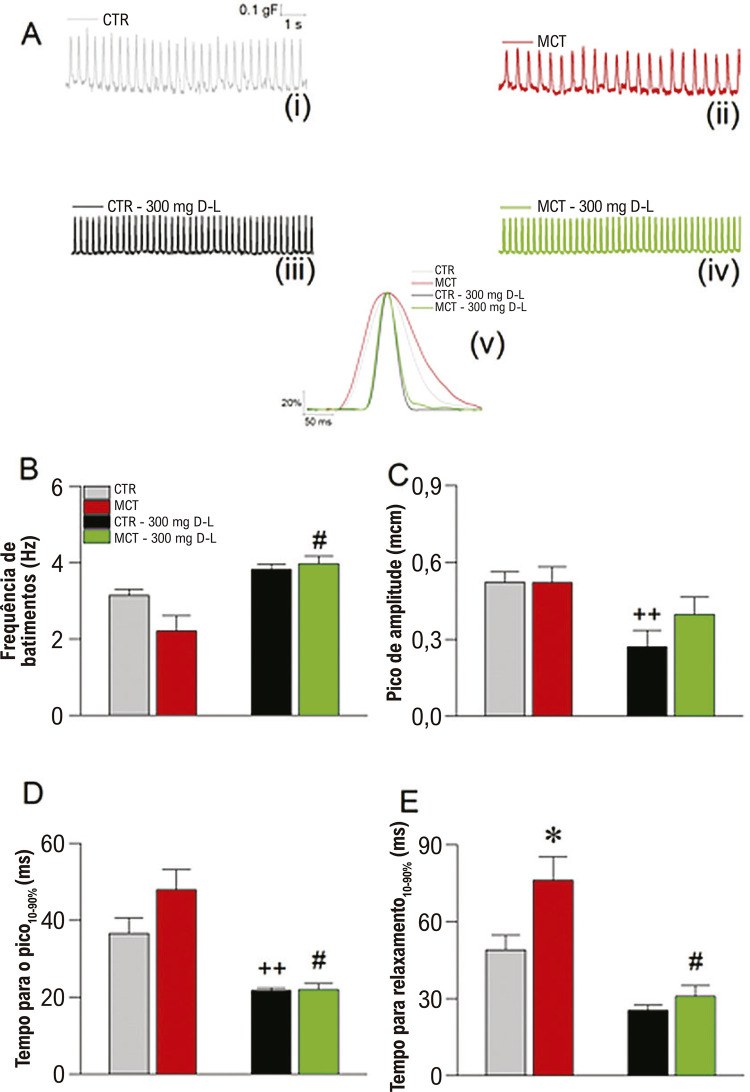
– Mudanças na contração atrial direita. (A) Traços representativos de contração espontânea do átrio direito para o grupo Controle (CTR) (i), animais tratados com Monocrotalina (MCT) (ii) animais CTR tratados com 300 mg/Kg/dia de D-Limoneno (D-L) (iii) e animais MCT tratados com 300 mg/Kg/dia de D-L (iv). Traçados normalizados sobrepostos para todos os grupos experimentais (v). (B) Frequência de batimentos. (C) Pico de amplitude. (D) Tempo para o pico de contração medido como o intervalo entre 10 e 90% para o desenvolvimento da contração. (E) Tempo para o relaxamento medido como o intervalo entre 10 e 90% para o desenvolvimento do relaxamento. Dados expressos como média ± erro padrão da média; p<0,05, ANOVA de uma via seguido do teste de Tukey, #MCT em comparação a MCT+D-L, ++ CTR em comparação a CTR+D-L, N=4.

Uma vez que o tratamento com D-L evitou o remodelamento da onda P, decidimos explorar a função mecânica do átrio direito e do átrio esquerdo. As (Figuras 5A-5E) resume nossos achados dos experimentos sobre contração atrial direita em uma frequência espontânea de batimento. Traçados representativos das contrações espontâneas do átrio direito em todos os grupos são apresentados na (Figura 5A, (i-iv). Uma curva de contração normalizada e sobreposta para todos os grupos está apresentada na (Figura 5A, v). A frequência de batimentos espontâneos mostrou uma tendência de redução no grupo MCT em comparação ao CTR (p=0,07). Um resultado interessante foi que a frequência no grupo MCT foi diferente que a observada no grupo MCT +D-L.

Não foi observada diferença no pico de amplitude nos grupos MCT e MCT+D-L em comparação a CTR e CTR+D-L. No entanto, ao se comparar CTR com CTR+D-L, houve uma atenuação significativa do pico de amplitude (p<0,05), e uma tendência similar, mas não significativa, ao se comparar MCT com MCT+D-L. Ainda, tecido do átrio direito de ratos tratados com MCT apresentou uma tendência não significativa para um tempo mais lento para o pico de contração em comparação ao CTR (Figura 5C). Por outro lado, o tratamento *in vivo* com D-L causou uma aceleração significativa (p<0,05) no tempo para o pico e para o relaxamento das curvas de contração do átrio direito em comparação entre CTR e CTR+D-L e entre MCT e MCT+D-L. Ainda, o tempo para o relaxamento foi mais lento no grupo MCT em comparação ao CTR (Figura 5E).

**Figura 6:**
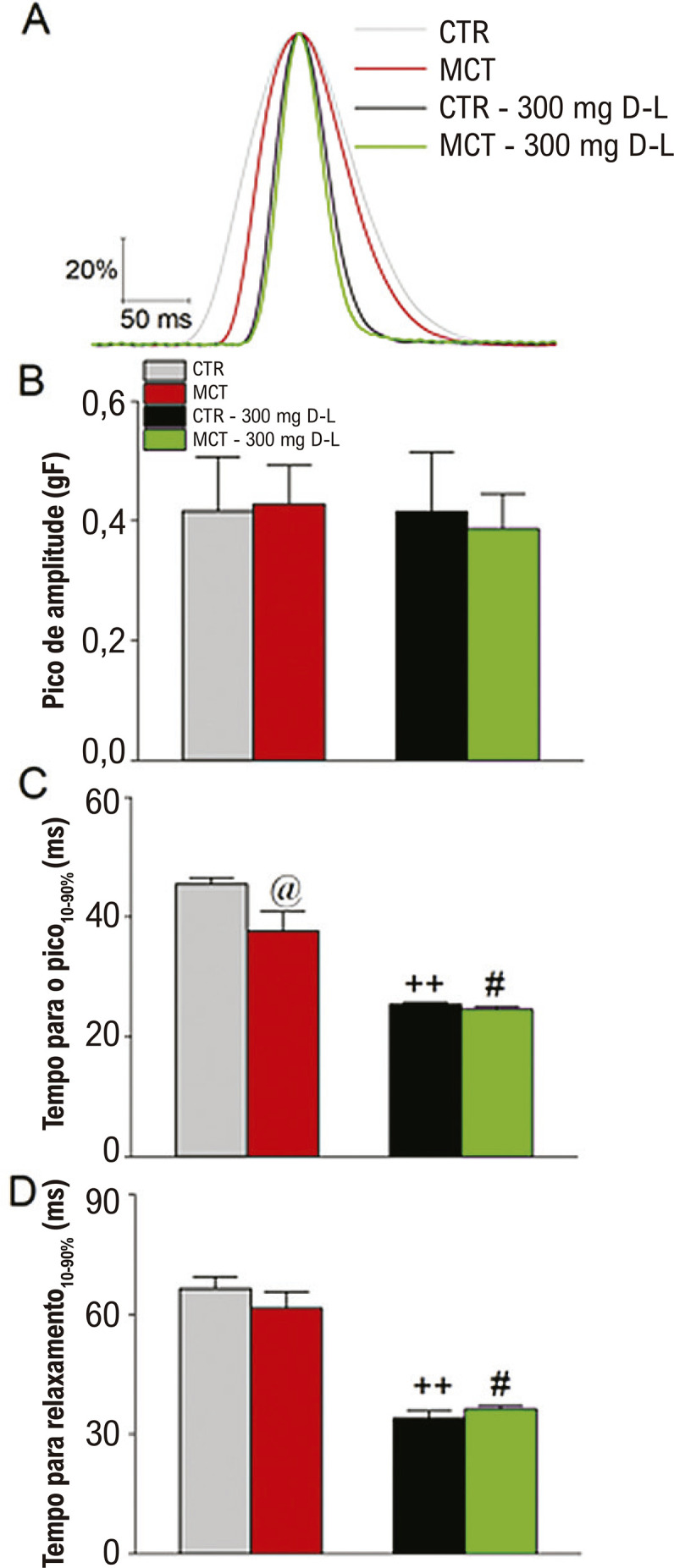
– Mudanças na contração atrial esquerda. (A) Traçados normalizados sobrepostos do átrio esquerdo sob frequência de estimulação de 3Hz para o grupo Controle (CTR), animais tratados com Monocrotalina (MCT) animais CTR tratados com 300 mg/Kg/dia de D-Limoneno (D-L) e animais MCT tratados com 300 mg/Kg/dia de D-L. (B) Pico de amplitude. (C) Tempo para o pico de contração medido como o intervalo entre 10 e 90% para o desenvolvimento da contração. (D) Tempo para o relaxamento medido como o intervalo entre 10 e 90% para o desenvolvimento do relaxamento. Dados expressos como média ± erro padrão da média; p<0,05, ANOVA de uma via seguido do teste de Tukey, @ comparando grupo MCT a todos os grupos, #comparando CTR a MCT+D-L, ++comparando CTR a CTR+D-L, N=4.

A frequência de batimentos espontâneos do tecido atrial direito estava na faixa de 3Hz; assim, decidimos estudar as propriedades da contração do átrio esquerdo usando uma frequência de estimulação de 3Hz (Figura 6). Uma curva normalizada sobreposta está apresentada na (Figura 6A) para todos os grupos experimentais. A contração atrial esquerda nos ratos MCT mostrou um tempo mais rápido para o pico de contração em comparação ao CTR (Figura 6C), mas não se observou diferença no tempo de relaxamento (Figura 6D). Ainda, os tempos para o pico de contração e relaxamento não foram diferentes entre os grupos CTR+D-L e MCT+D-L. Contudo, tanto o tempo para contração como o tempo para relaxamento foram significativamente mais rápidos nos grupos CTR+D-L e MCT+D-L quando comparados aos grupos CTR e MCT, respectivamente, indicando que a administração *in vivo* de D-L modula a função atrial esquerda.

### D-L exerce efeito anti-inflamatório no modelo de rato HP-MCT

**Figura 7:**
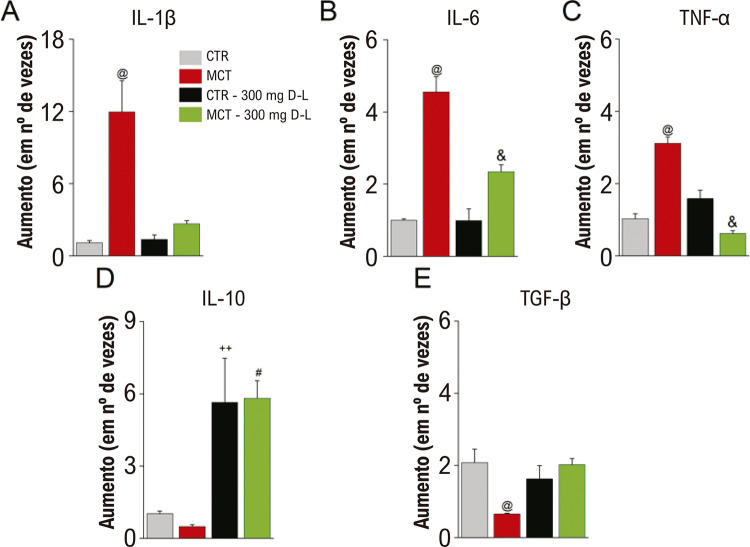
– Efeito do D-Limoneno (D-L) sobre a expressão de mRNA de citocinas pró-inflamatórias e citocinas anti-inflamatórias no tecido ventricular direito de ratos no modelo HP-MCT. As expressões de mRNA (100ng) de IL-1β (A), IL-6 (B), TNF-α (C), IL-10 (D) e TGF-β (G) foram analisadas por PCR quantitativo em Tempo real (RT-qPCR). Os resultados são expressos em média ± erro padrão da média; p<0,05, ANOVA de uma via seguido do teste de Tukey, @ comparando grupo MCT a todos os grupos, #comparando MCT a MCT+D-L, &comparando CTR+D-L a MCT-D+L, ++ comparando CTR a CTR+D-L (N = 4 animais por grupo em duplicata).

O MCT é conhecido por induzir inflamação no coração,^12^ o que pode ter um impacto na função e na estrutura do coração.^26^ Por isso, decidimos avaliar citoquinas inflamatórias chaves que estão alteradas na HP-MCT em ratos. A análise por RT-qPCR do tecido ventricular direito revelou que o grupo MCT apresentou uma expressão mais alta de citocinas pró-inflamatórias (IL-1β, IL-6 e TNF-α) (Figuras 7A-7C) em comparação ao CTR, e o tratamento com D-L preveniu a expressão exacerbada de todas as citocinas pró-inflamatórias testadas, restaurando seus níveis normais de expressão em comparação ao grupo CTR. Em contraste, a expressão das citocinas anti-inflamatórias IL-10 e TGF-β (Figuras 7D e 7E) estava atenuada no grupo MCT em comparação aos grupos CTR, CTR+D-L e MCT+D-L, e a administração *in vivo* de D-L melhorou seus níveis na HP-MCT.

## Discussão

No presente estudo, a administração de MCT (50 mg/Kg) em ratos jovens causou remodelamento mecânico elétrico e estrutural grave do coração, avaliado por achados histológicos, eletrocardiográficos e achados isolados de contração dos átrios. Isso é consistente com estudos prévios usando o modelo HP-MCT.^7-11^ Resultados de estudo prévio^23^ descreveram que a administração *in vivo* de D-L melhorou alguns aspectos fibróticos do remodelamento no modelo HP-MCT.

Na HP, a lesão às pequenas artérias pulmonares inclui danos às células endoteliais, espasmo arterial, fibrose e oclusão, e a inflamação é o principal contribuinte.^11,12^ Os danos vasculares podem levar à disfunção ventricular direita e à insuficiência cardíaca.^26^ À medida que a doença progride, a contratilidade anormal no lado direito do coração leva a uma superprodução de espécies reativas de oxigênio e de mediadores inflamatórios no coração.^12,13^ Assim, é intuitivo assumir que a administração de moléculas antioxidantes e anti-inflamatórias pode melhorar a função cardíaca na HP. Acredita-se que os processos inflamatórios exerçam um papel relevante na HP no homem e em experimentos.^1-3,12^

Pesquisa prévia mostrou que o tratamento oral com D-L (400 mg/Kg/dia) por três semanas consecutivas após a administração de MCT (60 mg/Kg) reduziu o remodelamento estrutural do coração, conforme avaliado pelo índice de Fulton.^23^ No entanto, há uma divergência nos resultados do índice de Fulton em nosso estudo, o que pode ser explicado em parte por: I) as linhagens dos animais eram diferentes entre os estudos. Ratos Wistar e Sprague-Dawley respondem de maneira diferente à lesão cardiovascular;^27^ II) ratos jovens e doses mais baixas de MCT influenciam a evolução do remodelamento cardíaco neste modelo experimental.^28,29^

Em nosso estudo, observamos que a administração de D-L atenuou o peso do ventrículo direito normalizado pelo comprimento da tíbia, indicando que a reversão não observada do índice de Fulton pode ser atribuído a um efeito distinto sobre o ventrículo direito e esquerdo (septo). Além disso, o D-L atenuou a fibrose cardíaca atenuada em ambos os ventrículos, o que contribui para a melhora da função ventricular *in vivo*.

Isso é corroborado pela observação de que o peso dos átrios direitos, que geralmente encontra-se aumentado neste modelo, foi revertido pela administração de D-L, o que não ocorreu com o peso dos átrios esquerdos. O remodelamento dos átrios direitos e esquerdos contribui para a arritmia atrial neste modelo experimental^7-10^ e, sendo assim, o D-L tem um efeito benéfico nesse aspecto. Tal fato é sustentado pela análise eletrocardiográfica. Ainda, observou-se que o remodelamento significativo no intervalo Qt não foi revertido pelo D-L, sugerindo que o monoterpeno pode exercer uma habilidade única de modular regiões distintas do coração.

A inflamação é um processo patológico essencial no remodelamento do coração que ocorre no modelo de HP-MCT em ratos.^11,12^ Assim, medimos a expressão das principais citocinas envolvidas na fisiopatologia de doenças cardíacas.^11,12^ Consistente com estudos prévios,^11,12^ nós demonstramos que a expressão de citocinas inflamatórias (IL-1β, IL-6 e TNF-α) está aumentada no grupo MCT, ao passo que a de citocinas anti-inflamatórias IL-10 e TGF-β está reduzida. D-L exerceu atividade anti-inflamatória ao restaurar os níveis de expressão de citocinas inflamatórias, aumentando o nível de IL-10 e restaurando a expressão de TGF-β.

A IL-10 é uma citocina anti-inflamatória versátil que exerce um papel crucial na regulação de vários aspectos da resposta imune, inflamação, propriedades vasculoprotetoras, e do remodelamento do tecido. A IL-10 é produzida principalmente por linfócitos Th2 durante a inflamação, em que atua suprimindo a produção de várias citocinas pró-inflamatórias.^30^ A IL-10 ganhou atenção significativa por sua capacidade de suprimir vasculopatia proliferativa e inflamatória.^30^ A IL-10 inibe inflamação diminuindo a produção das citocinas inflamatórias, exercendo, assim, feitos anti-inflamatórios. Além disso, ativa vias de sinalização que melhoram a expressão de genes anti-inflamatórios.^31^

O processo patológico da HP é caracterizado por níveis séricos anormais de citocinas pró-inflamatórias como IL-1 e IL-6.^32^ Pacientes com HP geralmente exibem níveis séricos basais elevados de IL-10, sugerindo uma resposta anti-inflamatória protetora à lesão presente.^33^ Dessa forma, a IL-10 tornou-se um ponto central em terapias potenciais cujo objetivo é combater fibrose associada à inflamação.^34^

A IL-10 exerce um papel indireto, mas crucial, em limitar a lesão cardíaca e fibrose. Sua via de sinalização, particularmente por meio de STAT3, promove o recrutamento e a retenção de células progenitoras endoteliais derivadas da medula óssea no local da lesão cardíaca, influenciando, assim, o reparo e a regeneração.^35,36^ Muitos dados da literatura sugerem um papel benéfico da IL-10 na HP. Por exemplo, no modelo de HP-MCT em ratos, a administração endovenosa de IL-10 via vetor adenoviral melhorou significativamente as taxas de sobrevida e reduziu a média de pressão arterial pulmonar.^30^ Ainda, a administração de IL-10 recombinante melhorou a função ventricular, reduziu o remodelamento hipertrófico, atenuou a fibrose cardíaca e a vasculopatia proliferativa, e diminuiu a taxa de mortalidade.^37^

A expressão sistêmica de IL-10 também melhorou a sobrevida de ratos no modelo HP-MCT, preveniu o desenvolvimento de hipertrofia ventricular direita, hipertrofia medial da artéria pulmonar, reduziu o acúmulo de macrófagos, a proliferação celular vascular, e reduziu os níveis de TGF-β1 e IL-6 no tecido pulmonar, o que é crítico na progressão de HP.^38^ Consistente com estudos prévios usando modelo de HP-MCT em ratos, um aumento nos níveis de IL-10, induzido por administração específica de IL-10, inibiu significativamente a expressão de IL-1β, IL-6 e TNF-α no ventrículo direito, e atenuou fibrose em ambos os ventrículos direito e esquerdo.^30^ Em geral, a IL-10 modula a dinâmica da rede de citocinas envolvida no remodelamento cardíaco induzido por HP, exercendo potencialmente seus efeitos em vários locais.

Nossos resultados evidenciaram que o D-L aumentou a expressão de IL-10 no tecido do ventrículo direito. A IL-10 pode estimular a síntese de anticitocinas endógenas e inibir receptores de citocinas pró-inflamatórias, e apresenta propriedades anti-inflamatórias potentes, reprimindo a expressão de IL-1β, IL-6 e TNF-α por macrófagos ativados.^39^ Um estudo prévio demonstrou que a IL-10, administrada por injeção intramuscular de um vetor viral adeno-associado, exerce vários efeitos preventivos sobre o remodelamento vascular proliferativo e inflamatório na HP, tais como diminuindo o acúmulo de macrófagos, a proliferação celular vascular, e os níveis de TGF-1 e IL-6 no tecido pulmonar.^30^ Por fim, já foi descrita a capacidade de o D-L modular a reposta inflamatória no coração.^40^

Em concordância com nossos resultados, um estudo prévio^12^ relatou que a inflamação esteve presente na evolução da HP e os níveis de mediadores inflamatórios estão aumentados durante a progressão da fase aguda à fase crônica da doença.^12^ A IL-1β é uma das primeiras citocinas a se elevarem no modelo experimental de MCT em ratos, e ela está relacionada a arritmias atriais e ventriculares em várias doenças cardíacas.^41,42^

O D-L pode aliviar a lesão cardíaca induzida por intoxicação por CCl_4_ por meio de seu potencial antioxidante e anti-inflamatório.^40^Além disso, as propriedades anti-inflamatórias do D-L foram demonstradas anteriormente por meio da inibição de NF-κB dependente de redox e de outras citocinas presentes na cascata inflamatória.^40^

Nesse contexto, outros estudos já relataram que o D-L modula a resposta inflamatória do coração.^40^ Em nosso estudo, apresentamos, pela primeira vez, uma análise mais específica e detalhada mostrando que a administração in vivo de D-L é capaz de modular especificamente o status inflamatório do ventrículo direito, que é afetado no remodelamento cardíaco induzido por HP-MCT.

## Conclusão

Com base nos resultados, pode-se concluir que a administração *in vivo* de D-L pode reduzir a formação de fibrose tecidual no modelo de PH-MCT em ratos jovens, como resumido na Figura Central. Além disso, o D-L restaura as mudanças eletrocardiográficas, e aumenta a expressão de citocinas anti-inflamatórias. No geral, o D-L pode ser um promissor agente anti-inflamatório contra a HP e a disfunção cardíaca.

## References

[1] Humbert M, Kovacs G, Hoeper MM, Badagliacca R, Berger RMF, Brida M (2022). 2022 ESC/ERS Guidelines for the Diagnosis and Treatment of Pulmonary Hypertension. Eur Heart J.

[2] Vonk-Noordegraaf A, Haddad F, Chin KM, Forfia PR, Kawut SM, Lumens J (2013). Right Heart Adaptation to Pulmonary Arterial Hypertension: Physiology and Pathobiology. J Am Coll Cardiol.

[3] Gelzinis TA (2022). Pulmonary Hypertension in 2021: Part I-Definition, Classification, Pathophysiology, and Presentation. J Cardiothorac Vasc Anesth.

[4] Boucherat O, Agrawal V, Lawrie A, Bonnet S (2022). The Latest in Animal Models of Pulmonary Hypertension and Right Ventricular Failure. Circ Res.

[5] Fingrova Z, Ambroz D, Jansa P, Kuchar J, Lindner J, Kunstyr J (2021). The Prevalence and Clinical Outcome of Supraventricular Tachycardia in Different Etiologies of Pulmonary Hypertension. PLoS One.

[6] Andersen MØ, Diederichsen SZ, Svendsen JH, Carlsen J (2021). Assessment of Cardiac Arrhythmias Using Long-term Continuous Monitoring in Patients with Pulmonary Hypertension. Int J Cardiol.

[7] Teixeira-Fonseca JL, Joviano-Santos JV, Alcântara FS, Conceição MRL, Leal-Silva P, Roman-Campos D (2023). Evaluation of Right Atrium Structure and Function in a Rat Model of Monocrotaline-induced Pulmonary Hypertension: Exploring the Possible Antiarrhythmic Properties of Amiodarone. Clin Exp Pharmacol Physiol.

[8] Teixeira-Fonseca JL, Joviano-Santos JV, Beserra SS, Conceição MRl, Leal-Silva P, Marques LP (2023). Exploring the Involvement of TASK-1 in the Control of Isolated Rat Right Atrium Function from Healthy Animals and an Experimental Model of Monocrotaline-induced Pulmonary Hypertension. Naunyn Schmiedebergs Arch Pharmacol.

[9] Teixeira-Fonseca JL, Conceição MRL, Leal-Silva P, Roman-Campos D (2023). Ranolazine Exerts Atrial Antiarrhythmic Effects in a rat Model of Monocrotaline-induced Pulmonary Hypertension. Basic Clin Pharmacol Toxicol.

[10] Teixeira-Fonseca JL, Joviano-Santos JV, Machado FS, Silva PLD, Conceição MRL, Roman-Campos D (2023). Isolated Left Atrium Morphofunctional Study of an Experimental Pulmonary Hypertension Model in Rats. Arq Bras Cardiol.

[11] Hiram R, Naud P, Xiong F, Al-U'datt D, Algalarrondo V, Sirois MG (2019). Right Atrial Mechanisms of Atrial Fibrillation in a Rat Model of Right Heart Disease. J Am Coll Cardiol.

[12] Tang C, Luo Y, Li S, Huang B, Xu S, Li L (2021). Characteristics of Inflammation Process in Monocrotaline-induced Pulmonary Arterial Hypertension in Rats. Biomed Pharmacother.

[13] Redout EM, Wagner MJ, Zuidwijk MJ, Boer C, Musters RJ, van Hardeveld C (2007). Right-ventricular Failure is Associated with Increased Mitochondrial Complex II Activity and Production of Reactive Oxygen Species. Cardiovasc Res.

[14] Csiszar A, Labinskyy N, Olson S, Pinto JT, Gupte S, Wu JM (2009). Resveratrol Prevents Monocrotaline-induced Pulmonary Hypertension in Rats. Hypertension.

[15] Nie X, Wu Z, Shang J, Zhu L, Liu Y, Qi Y (2023). Curcumol Suppresses Endothelial-to-mesenchymal Transition via inhibiting the AKT/GSK3ß Signaling Pathway and Alleviates Pulmonary Arterial Hypertension in Rats. Eur J Pharmacol.

[16] Benoist D, Stones R, Drinkhill M, Bernus O, White E (2011). Arrhythmogenic Substrate in Hearts of Rats with Monocrotaline-induced Pulmonary Hypertension and Right Ventricular Hypertrophy. Am J Physiol Heart Circ Physiol.

[17] Spaczynska M, Rocha SF, Oliver E (2020). Pharmacology of Pulmonary Arterial Hypertension: An Overview of Current and Emerging Therapies. ACS Pharmacol Transl Sci.

[18] O'Callaghan DS, Humbert M (2012). A Critical Analysis of Survival in Pulmonary Arterial Hypertension. Eur Respir Rev.

[19] Erasto P, Viljoen AM (2008). Limonene - A Review: Biosynthetic, Ecological and Pharmacological Relevance. Nat Prod Commun.

[20] Ravichandran C, Badgujar PC, Gundev P, Upadhyay A (2018). Review of Toxicological Assessment of d-limonene, a Food and Cosmetics Additive. Food Chem Toxicol.

[21] Ramos CAF, Sá RCDS, Alves MF, Benedito RB, Sousa DP, Diniz MFFM (2015). Histopathological and Biochemical Assessment of d-limonene-induced Liver Injury in Rats. Toxicol Rep.

[22] Silva EAP, Santos DM, Carvalho FO, Menezes IAC, Barreto AS, Souza DS (2021). Monoterpenes and Their Derivatives as Agents for Cardiovascular Disease Management: A Systematic Review and Meta-analysis. Phytomedicine.

[23] Touvay C, Vilain B, Carré C, Mencia-Huerta JM, Braquet P (1995). Effect of Limonene and Sobrerol on Monocrotaline-induced Lung Alterations and Pulmonary Hypertension. Int Arch Allergy Immunol.

[24] Rocchetti M, Sala L, Rizzetto R, Staszewsky LI, Alemanni M, Zambelli V (2014). Ranolazine Prevents INaL Enhancement and Blunts Myocardial Remodelling in a Model of Pulmonary Hypertension. Cardiovasc Res.

[25] Costa HHM, Bielavsky M, Orts DJB, Araujo S, Adriani PP, Nogueira JS (2023). Production of Recombinant Zika Virus Envelope Protein by Airlift Bioreactor as a New Subunit Vaccine Platform. Int J Mol Sci.

[26] Humbert M, Lau EM, Montani D, Jaïs X, Sitbon O, Simonneau G (2014). Advances in Therapeutic Interventions for Patients with Pulmonary Arterial Hypertension. Circulation.

[27] Ebmeyer U, Keilhoff G, Wolf G, Röse W (2002). Strain Specific Differences in a Cardio-pulmonary Resuscitation Rat Model. Resuscitation.

[28] Steven S, Oelze M, Brandt M, Ullmann E, Kröller-Schön S, Heeren T (2017). Pentaerythritol Tetranitrate In Vivo Treatment Improves Oxidative Stress and Vascular Dysfunction by Suppression of Endothelin-1 Signaling in Monocrotaline-induced Pulmonary Hypertension. Oxid Med Cell Longev.

[29] Kawade A, Yamamura A, Fujiwara M, Kobayashi S, Mori S, Horii C (2021). Comparative Analysis of Age in Monocrotaline-induced Pulmonary Hypertensive Rats. J Pharmacol Sci.

[30] Ito T, Okada T, Miyashita H, Nomoto T, Nonaka-Sarukawa M, Uchibori R (2007). Interleukin-10 Expression Mediated by an Adeno-associated Virus Vector Prevents Monocrotaline-induced Pulmonary Arterial Hypertension in Rats. Circ Res.

[31] Fledderus JO, Joles JA (2011). Expanding the Beneficial Pleiotropic Repertoire of Interleukin-10. J Hypertens.

[32] Humbert M, Monti G, Brenot F, Sitbon O, Portier A, Grangeot-Keros L (1995). Increased Interleukin-1 and Interleukin-6 Serum Concentrations in Severe Primary Pulmonary Hypertension. Am J Respir Crit Care Med.

[33] Groth A, Vrugt B, Brock M, Speich R, Ulrich S, Huber LC (2014). Inflammatory Cytokines in Pulmonary Hypertension. Respir Res.

[34] Steen EH, Wang X, Balaji S, Butte MJ, Bollyky PL, Keswani SG (2020). The Role of the Anti-inflammatory Cytokine Interleukin-10 in Tissue Fibrosis. Adv Wound Care.

[35] Krishnamurthy P, Thal M, Verma S, Hoxha E, Lambers E, Ramirez V (2011). Interleukin-10 Deficiency Impairs Bone Marrow-derived Endothelial Progenitor Cell Survival and Function in Ischemic Myocardium. Circ Res.

[36] Rajasingh J, Thangavel J, Siddiqui MR, Gomes I, Gao XP, Kishore R (2011). Improvement of Cardiac Function in Mouse Myocardial Infarction after Transplantation of Epigenetically-modified Bone Marrow Progenitor Cells. PLoS One.

[37] Verma SK, Krishnamurthy P, Barefield D, Singh N, Gupta R, Lambers E (2012). Interleukin-10 Treatment Attenuates Pressure Overload-induced Hypertrophic Remodeling and Improves Heart Function via Signal Transducers and Activators of Transcription 3-dependent Inhibition of Nuclear factor-?B. Circulation.

[38] Chen S, Kapturczak MH, Wasserfall C, Glushakova OY, Campbell-Thompson M, Deshane JS (2005). Interleukin 10 Attenuates Neointimal Proliferation and Inflammation in Aortic Allografts by a Heme Oxygenase-dependent Pathway. Proc Natl Acad Sci USA.

[39] Zhang JM, An J (2007). Cytokines, Inflammation, and Pain. Int Anesthesiol Clin.

[40] AlSaffar RM, Rashid S, Ahmad SB, Rehman MU, Hussain I, Ahmad SP (2022). D-limonene (5 (one-methyl-four-[1-methylethenyl]) cyclohexane) Diminishes CCl4-induced Cardiac Toxicity by Alleviating Oxidative Stress, Inflammatory and Cardiac Markers. Redox Rep.

[41] Monnerat G, Alarcón ML, Vasconcellos LR, Hochman-Mendez C, Brasil G, Bassani RA (2016). Macrophage-dependent IL-1ß Production Induces Cardiac Arrhythmias in Diabetic Mice. Nat Commun.

[42] Matsushita N, Ishida N, Ibi M, Saito M, Takahashi M, Taniguchi S (2019). IL-1ß Plays an Important Role in Pressure Overload-Induced Atrial Fibrillation in Mice. Biol Pharm Bull.

