# Supplementary material for: Atividade Anti-Inflamatória In Vivo do D-Limoneno em um Modelo de Hipertensão Pulmonar Induzida por Monocrotalina em Ratos: Implicações à Função Cardíaca
Source: Arq Bras Cardiol. 2024 Nov 22;121(12):e20240195. [Article in Portuguese] doi: 10.36660/abc.20240195 (PMC11634294; doi:10.36660/abc.20240195)
Supplement: Translation

# Translation: In Vivo Anti-Inflammatory Activity of D-Limonene in a Rat Model of Monocrotaline-Induced Pulmonary Hypertension: Implications to the Heart Function

**DOI:** 10.36660/abc.20240195i

**Authors:** Jorge Lucas Teixeira-Fonseca, Diego Jose Belato y Orts, Polyana Leal da Silva, Michael Ramon de Lima Conceição, Hernan Hermes, Carlos R. Prudencio, Danilo Roman-Campos

**Affiliations:** 1 Brazil Universidade Federal de São Paulo, São Paulo, SP – Brazil; 2 Brazil Instituto Adolfo Lutz, São Paulo, SP – Brazil; 3 Brazil Universidade São Paulo, São Paulo, SP – Brazil

**Keywords:** Pulmonary Hypertension, Anti-Inflammatory Agents, Biological Products

## Abstract

**Background:**

D-limonene (D-L) is the major monocyclic monoterpene in citrus plants with anti-inflammatory properties. Pulmonary hypertension (PH) can cause right heart dysfunction and increases the risk of death, partially due to inflammatory response in the heart.

**Objective:**

To evaluate the possible protective effect of D-L on cardiac function in a rat model of monocrotaline-induced PH (MCT-PH).

**Methods:**

Electrocardiogram was monitored in vivo. Masson Trichrome technique was deployed to verify fibrosis in the heart. Contractility function of isolated atrial tissue was studied using organ bath chamber. Real-time quantitative PCR was applied to quantify inflammation in the right ventricle.

**Results:**

The MCT-PH group showed electrical and structural heart remodeling, with the presence of fibrosis in the cardiac tissue and in vivo electrocardiographic changes. Treatment with D-L partially prevented the development of tissue fibrosis and the increase in P wave duration in the MCT-PH group. The contraction and relaxation velocity of isolated right and left atrium were accelerated in CTR and MCT-PH animals treated with D-L. Finally, D-L was able to prevent the abnormal expression of the key inflammatory cytokines (interleukin 1-β, interleukin 6 and tumor necrosis factor-α) in the right ventricle of MCT-PH animals. D-L was able to enhance the production of the anti-inflammatory cytokine Interleukin-10.

**Conclusion:**

Our results showed that in vivo administration of D-L partially prevented the molecular, structural and functional remodeling of the heart in the MCT-PH model with attenuation of the inflammatory response in the heart.

## Introduction

Pulmonary hypertension (PH) is a chronic and multifactorial disease, characterized by progressive pulmonary arterial bed remodeling, resulting in endothelial cell dysfunction, abnormal proliferation of pulmonary artery smooth muscle cells and the presence of inflammatory cells in the tunica adventitia.^1^ These changes together lead to an increase in pulmonary vascular resistance and mean pulmonary arterial pressure.^2^ In more advanced stages of the PH, pressure overload in the right ventricle may occur, contributing to hypertrophy and dilation of the right heart and, eventually failure to maintain cardiac output.^3^

Monocrotaline (MCT)-induced PH (MCT-PH) in rats is the main preclinical model used in understanding the pathophysiology of human PH.^4^ MCT-PH recapitulates several right heart features observed in patients with compromised cardiac function secondary to PH, including electrical and structural remodeling of the myocardium.^1,5,6^

During the time course of PH, injuries to the myocardium compromise the electrical conduction system, with a risk for the emergence of arrhythmias, both in humans^5,6^ and in the MCT-PH model in rats.^7-10^ Previous studies suggested that apoptotic, oxidative and inflammatory pathways are important players in the pathogenesis of atrial and ventricular heart disease observed in the MCT-PH.^11-16^ Despite significant advances in pharmacological therapies^17^ and clinical management of the disease, the survivability of patients with PH remains low.^18^ Therefore, it is important to develop new pharmacological therapies that aim to reduce electromechanical remodeling of the heart secondary to PH.

The D-Limonene (4-isopropenyl-1-methylcyclohexene), or D-L, is a monocyclic monoterpene, predominantly found in oils extracted from the peel of citrus fruits, such as lemon, orange and mandarin.^19^ Studies have shown that D-L has anti-inflammatory and antioxidant therapeutic properties.^20-22^ Furthermore, D-L was able to attenuate some morphological remodeling of the heart in the rat model of MCT-PH,^23^ but the authors did not evaluate cardiac function or the possible cardioprotective mechanism involved. In this sense, we speculate that D-L could reduce cardiac tissue damage and altered heart function induced by MCT-PH.

Thus, the objective of this study was to verify whether *in vivo* administration of D-L can reduce the inflammatory response in the heart and prevent the development of functional and structural remodeling of the cardiac tissue after administration of MCT in rats.

## Methods

### Animals

All animal handling procedures were approved by the Ethics Committee for Animal Use (CEUA) of the Federal University of São Paulo (#5438060923). The rats weighing ~100g were obtained from the Center for Development of Experimental Models of Biology and Medicine (CEDEME) and housed in institutional animal care facilities on a 12-hour light/dark cycle with food and water *ad libitum*.

### Experimental design

The rats were randomly divided into four groups, as follows: 1) control group (CTR): the rats were treated with 1ml/Kg/day of corn oil (vehicle); 2) MCT group: rats received a single dose of MCT 50 mg/Kg (SIGMA Chemical Co. St. Louis, MO, USA), via intraperitoneal injection (i.p.);^7-10^ 3) CTR+D-L: rats received 300 mg/Kg/day of D-L (SIGMA Chemical Co. St. Louis, MO. USA) diluted in corn oil; 4) MCT+D-L: after treatment with MCT (50mg/Kg, i.p.), rats received 300 mg/Kg/day of D-L diluted in corn oil. Vehicle and D-L treatments were administered orally (gavage) during 18 to 20 consecutive days, two days after MCT administration. To avoid interference with MCT metabolism, D-L treatment was started two days after MCT injection.^24^

### Experiments in isolated right and left atria

The right and left atria were cut perpendicularly and mounted in a tank for isolated organs containing Tyrode’s solution (in mM): 140 NaCl, 5.4 KCl, 1.8 CaCl_2_, 1.0 MgCl_2_, 0.33 NaH_2_PO_4_, 11 Dextrose and 5 HEPES that was continuously gasified with 99.9% O_2_. The ends of the atrial were suspended horizontally by stainless steel hooks and equilibrated under a resting tension of 0.5 gf (4.9 mN) for at least 40 minutes. Only the left atrium was subjected to electrical field stimulation (3 Hz, 100 V, 0.5 ms). Atrial contraction was normalized using OriginLab software.

### Histopathological examination

The hearts were removed from the thoracic cavity and washed with phosphate-buffered saline (PBS). Afterwards they were fixed with 4% paraformaldehyde for 24 hours, and then dehydrated in ethanol, cleared in xylene and embedded in paraffin. Paraffin-embedded tissue blocks were sectioned at 5 μm thickness and deparaffinized by submersion in xylene, followed by rehydration with alcohol. The sections were stained with Masson Trichrome, according to the manufacturer’s instructions. After staining, images were acquired using an optical microscope (Leica DM4000B; Leica Microsystems). The area of myocardial fibrosis was quantified using Image J software (NIH, Bethesda, MD, USA).^8^

### Surface Electrocardiography

The rats were sedated with 1.5-2.0% isoflurane (Isoforine®, Cristália). The electrocardiographic examination was performed on ECG-PC version 2.07 ®-TEB before the treatments were started and on the last day. The lead II electrocardiogram was used to calculate P wave duration, QRS complex, PR intervals, QT interval and heart rate. QT interval values were corrected using the Bazett formula QTc=QT/√RR. All ECG tracings were analyzed offline. For each trait, measurements were taken from an average of 1 min of ECG recording.

### RNA extraction and Real-Time Quantitative PCR (RT-qPCR) analysis

Total RNA was extracted from rat right ventricular tissue using TRIzol LS reagent (Life Technologies, Paisley, UK) and cDNA was synthesized using the GoScript Reverse Transcription System (Promega, Dübendorf, Switzerland) according to the manufacturer’s instructions. Four animals from each group (CTR, MCT, CTR + D-L and MCT + D-L) were used for analysis of the cellular expression of interleukin-1β (IL-1β), interleukin-6 (IL-6), interleukin-10 (IL-10), tumor necrosis factor alpha (TNF-α), and the transforming growth factor beta (TGF-β) by real-time quantitative PCR (RT-qPCR) using specific primers^12^ and GoTaq qPCR Master Mix kit (Promega). Relative gene expression was normalized to endogenous expression of β-actin mRNA per each representative control.

RT-qPCR was performed on an ABI 7500 Fast real-time PCR system (Applied Biosystems, Waltham, MA) with the following conditions: initial denaturation (5 min 95°C); denaturation by 40 cycles × 15 sec 95°C, 60 sec 60°C primer annealing/elongation. The fluorescence was recorded during the annealing/elongation step in each cycle. We performed two technical replicates for each of the cytokines per group to evaluate the relative quantification (RQ).^25^

The RQ of a target gene in comparison with a reference gene was calculated according to the equation $\mathrm{RQ}\;=\;\frac{{{\mathrm{Efficiency}}_{\mathrm{target}}}^{∆\mathrm{Ct}\;\mathrm{target}\;(\mathrm{control}\;-\;\mathrm{sample}}}{{{\mathrm{Efficiency}}_{\mathrm{reference}}}^{∆\mathrm{Ct}\;\mathrm{reference}\;(\mathrm{control}\;-\;\mathrm{sample})}}$ .

### Statistical analysis

All data are presented as mean ± standard error of the mean (S.E.M.). The normality of the dependent variable was tested with the Shapiro-Wilk test. Comparisons between groups were performed using One-way or Two-way analysis of variance, and the multiple comparison Tukey post-test. The level of significance to reject the null hypothesis was p <0.05. The number of animals was represented by (N). Data were analyzed in Excel ® (Microsoft, USA) and Origin 8.0^®^ (OriginLab, USA).

## Results

In vivo D-L administration does not impede MCT-induced compromised body weight gain in young rats

MCT administration causes changes in the body weight gain in rats.^8^ Thus, the animals were weighted at the beginning and at the end of the experiment. An increase in body weight was observed in all groups at 20 days as compared with baseline (Figure 1) (p<0.05). However, at 20 days after MCT administration, the body weight of the MCT animals and the MCT+D-L animals were reduced compared to CTR and CTR+D-L, respectively.

**Figure 1:** – Time course of body weight. Body weight was measured in control (CTR), monocrotaline (MCT), CTR + 300 mg/kg/day of D-limonene (D-L), and MCT treated animals + 300 mg/kg/day of D-L on day 0 and on day 20. Data are expressed as mean ± S.E.M.; p<0.05, two way-ANOVA for repeated measurements followed by Tukey test, %comparing 0 days to 20 days within your respective control, *comparing CTR to MCT at 20 days, &comparing CTR 300 mg D-L to MCT 300 mg D-L at 20 days; N=4-6.

### Structural and electrical heart remodeling caused by MCT is partially prevented by D-L administration in young rats

Structural remodeling of the heart is a marker of MCT-PH in rats.^7-10^ Measurement of the weight of the heart, right ventricle, right atrium, left atrium, ratio between right ventricle and tibial length, and Fulton index showed significant increases in the MCT group when compared to the CTR (Figures 2A-2F). Also, the average weight of the left atrium and the Fulton index were higher in the MCT+D-L animals when compared to the CTR+D-L (Figures 2D and 2F).

**Figure 2:** – Morphological changes in the heart. Experimental groups are control (CTR), monocrotaline (MCT), CTR + 300 mg/kg/day of D-limonene (D-L), and MCT treated animals + 300 mg/kg/day of D-L. (A) Heart weight. (B) Right ventricle weight. (C) Right atrium weight. (D) Left atrium weight. (E) Normalized right ventricle weight by tibial length. (F) Fulton index. Data are expressed as mean ± S.E.M. p<0.05, One Way-ANOVA followed by Tukey test *comparing CTR to MCT and &comparing CTR+D-L to MCT + D-L. N=4-5.

It is well documented that increased heart fibrosis is commonly found in MCT treated animals.^11-16^ Thus, histological analyses were performed and the results for the right and left ventricles are illustrated in (Figure 3A). The MCT animals displayed enhanced fibrosis (Figures 3B and 3C) in both ventricles, while treatment with D-L attenuated fibrosis in both.

**Figure 3:** – D-limonene (D-L) attenuate fibrosis in the heart. Experimental groups are control (CTR), monocrotaline (MCT), CTR + 300 mg/kg/day of D-L, and MCT treated animals + 300 mg/kg/day of D-L on day 0 and on day 20. (A) representative section of the right ventricle and left ventricle. (B) Fibrosis in the right ventricle. (C) Fibrosis in the left ventricle. Data are expressed as mean ± S.E.M. p<0.05, one way-ANOVA followed by Tukey test, @comparing MCT to all other groups.

Next, we explored the ECG profile of the animals, which is summarized in (Figure 4). In (Figure 4A), i-iv show representative ECG tracings of the four experimental groups on day 20. As shown in (Figure 4B), no changes in the heart rate, measured as the RR interval, nor in the QRS complex were observed (Figure 4D). MCT administration caused increased P wave duration (Figure 4C), and *in vivo* D-L treatment prevented the phenotype (MCT+D-L group). Moreover, the QT interval increased in the MCT group when compared to the CTR group (Figure 4D), and *in vivo* D-L treatment did not prevent the lengthening in the QT interval.

**Figure 4:** – Electrocardiographic changes. (A) Representative traces of electrocardiogram lead II for controls (CTR) (i), and monocrotaline (MCT) (ii), CTR + 300 mg/kg/day of D-limonene (D-L) (iii), and MCT treated animals + 300 mg/kg/day of D-L on day 0 and on day 20 (iv). (B) RR interval. (C) P wave duration. (D) QRS Complex. (E) QT interval. Data are expressed as mean ± S.E.M. p<0.05, One Way-ANOVA followed by Tukey test, @comparing MCT to all groups, *comparing CTR to MCT and & comparing CTR+D-L to MCT+D-L. N=4-5.

### D-L modulates left atrial and right atrial function

Since D-L prevented the P wave remodeling we decided to explore the right atrial and left atrial mechanical function. Figures 5A-5E summarize our findings from the right atrial contraction experiments at spontaneous beating frequency. Representative traces of spontaneous contractions of the right atrium in all groups are shown in (Figure 5A, (i-iv), and a superimposed and normalized contraction curve for all groups is shown in (Figure 5A, v). Spontaneous beating frequency had a trend toward reduction in the MCT group compared to CTR (p=0.07). Interestingly, the frequency in the MCT was different from that in the MCT +D-L.

**Figure 5:** – Right atrial (RA) contraction changes. (A) Representative traces of RA spontaneous contraction for the CTR (i), monocrotaline (MCT) (ii) treated animals, CTR administered 300 mg/kg/day of D-L (iii) and MCT administered 300 mg/kg/day of D-limonene (iv). Superimposed normalized traces for all experimental groups (v). (B) Beating frequency. (C) Peak amplitude. (D) Time to peak of contraction measured as the interval between 10 and 90% to development of contraction. (E) Time to relaxation measured as the interval between 10 and 90% to development of relaxation. Data are expressed as mean ± S.E.M. p<0.05, One Way-ANOVA followed by Tukey test, #MCT compared with MCT+D-L, ++CTR compared with CTR+D-L; N=4.

There was no difference in the peak amplitude when comparing CTR to MCT and CTR+D-L to MCT+D-L (Figure 5C). However, when comparing CTR to CTR+D-L a significant attenuation of peak amplitude was found (p<0.05), and a similar trend, but not significant when comparing MCT to MCT+D-L. Also, right atrial tissue from MCT rats showed a non-significant trend towards a slower time to peak contraction when compared to CTR (Figure 5D). On contrary, *in vivo* treatment with D-L caused a significant speeding (p<0.05) in the time to peak and relaxation of right atrial contraction curves, when comparing CTR to CTR+D-L and MCT to MCT+D-L group. Also, the time to relaxation was slower in the MCT group when compared to the CTR (Figure 5E).

The spontaneous beating frequency of right atrial tissue was in the range of 3 Hz, thus we decided to further study the left atrial contraction properties using a pacing frequency of 3 Hz (Figure 6). A superimposed normalized curve is shown in (Figure 6A) for all experimental groups. There was no difference in the peak amplitude among all groups, (Figure 6B). Interestingly, left atrial contraction in MCT rats showed faster time to peak contraction when compared to CTR (Figure 6C), but no difference in the relaxation time was found, (Figure 6D). Also, the times to peak contraction and relaxation were not different between CTR+D-L and MCT+D-L. However, both time to contraction and time to relaxation were significantly faster in the CTR+D-L and in the MCT+D-L groups when compared to CTR and MCT, respectively, indicating that *in vivo* D-L administration modulates left atrial function.

**Figure 6:** – Left atrial (LA) contraction changes. (A) Representative superimposed normalized traces of LA paced at 3 Hz for the controls (CTR), monocrotaline (MCT) treated animals, CTR administered 300 mg/kg/day of D-limonene (D-L) and MCT administered 300 mg/Kg/day of D-L. (B) Peak amplitude. (C) Time to peak of contraction measured as the interval between 10 and 90% to development of contraction. (D) Time to relaxation measured as the interval between 10 and 90% to development of relaxation. Data are expressed as mean ± S.E.M. p<0.05, one Way-ANOVA followed by Tukey post-test, @comparing MCT to all other groups, #comparing MCT to MCT+D-L, ++comparing CTR to CTR+D-L, N=4|Z|.

### D-L display anti-inflammatory effect in MCT-PH rat model

MCT is known to induce heart inflammation,^12^ which may impact the function and structure of the heart.^26^ Thus, we decided to evaluate key inflammatory cytokines known to be altered in the course of MCT-PH in rats. The RT-qPCR analysis of the right ventricular tissue revealed that MCT group had a higher expression of pro-inflammatory cytokines (IL-1β, IL-6 and TNF-α) (Figures 7A-7C), in comparison to CTR, and treatment with D-L prevented the overexpression of all pro-inflammatory cytokines tested, restoring their normal expression levels when comparing to CTR group. On the contrary, the expression of anti-inflammatory cytokines IL-10 and TGF-β (Figures 7D and 7E) was found attenuated in the MCT group when compared to CTR, CTR+D-L and MCT+D-L groups, and *in vivo* D-L administration improved their levels in the MCT-PH.

**Figure 7:** – Effect of D-limonene (D-L) on the mRNA expression of pro-inflammatory and anti-inflammatory cytokines in right ventricular tissue of MCT-PH model rats. The mRNA (100 ng) expressions of IL-1β (A), IL-6 (B), TNF-α (C), IL-10 (D) and TGF-β (G) were analyzed by real time-qPCR. The results are reported as the mean ± S.E.M. p<0.05, One Way-ANOVA followed by Tukey post-test. @comparing MCT to all other groups, #comparing MCT to MCT+D-L, &comparing CTR+D-L to MCT-D+L, ++ comparing CTR to CTR+D-L (N = 4 animals per group in duplicate).

## Discussion

In the present study, the administration of MCT (50 mg/kg) in young rats caused severe structural, electrical and mechanical remodeling of the heart, assessed through histological, electrocardiographic and isolated atria contraction findings. This is consistent with previous studies using the MCT-PH model.^7-11^ Previous findings^23^ described that *in vivo* D-L administration improved some fibrotic aspects of tissue remodeling in the MCT-PH model.

In PH, damage to the small pulmonary arteries includes impairment of endothelial cells, artery spasm, fibrosis and occlusion, and inflammation is the major contributor.^11,12^ The vascular changes can lead to right ventricular dysfunction and heart failure.^26^ As the disease progress, abnormal contractility in the right side of the heart leads to an overproduction of reactive oxygen species and inflammatory mediators in the heart.^12,13^ Thus, it is intuitive to assume that the administration of antioxidant and anti-inflammatory molecules can improve cardiac function in PH. It is believed that inflammatory processes play a relevant role in human and experimental PH.^1-3,12^

Previous research has shown that oral treatment with D-L (400 mg/Kg/day) for three consecutive weeks after MCT administration (60 mg/kg) reduced the structural remodeling of the heart, as assessed by the Fulton index.^23^ However, there is a divergence in the results of the Fulton index observed in our study, which can be explained in part by: I) the animal strains were different between studies. Wistar and Sprague-Dawley rats are known to have distinct responses to cardiovascular injury;^27^ II) Young rats and lower doses of MCT influence the course of heart remodeling in this experimental model.^28,29^

In our study we observed that D-L administration attenuated right ventricular weight normalized by tibial length, indicating that the unobserved reversal of the Fulton index may be due to a distinct effect on the right and left ventricle (septum). Furthermore, D-L attenuated the heart fibrosis observed in both the right and left ventricles, which contributes to the improvement of ventricular function *in vivo*.

This is supported by the observation that the weight of the right atria, which is usually increased in this model, was reversed by D-L administration, but not the left atria weight gain. Remodeling of both, right and left atria contributes to atrial arrhythmia in this experimental model,^7-10^ thus D-L is beneficial in this regards. This aspect is further supported by the ECG analysis. Also, it was found that the significant remodeling observed in the QT interval was not reversed by D-L, suggesting that the monoterpene may have a unique ability to modulate distinct regions of the heart.

Inflammation is an essential pathological process involved in heart remodeling in the MCT-PH rat model.^11,12^ Thus, we measured the expression of the main cytokines involved in the pathophysiology of heart diseases.^11,12^ Consistent with previous studies,^11,12^ we demonstrated that the expression level of inflammatory cytokines (IL-1β, IL-6 and TNF-α) is enhanced in the MCT group, while the anti-inflammatory cytokines IL-10 and TGF-β are down-regulated. D-L displayed anti-inflammatory activity through restoring the expression levels of inflammatory cytokines, increasing the level of IL-10 and restoring the TGF-β expression.

Il-10 is a versatile anti-inflammatory cytokine that plays a crucial role in regulating various aspects of the immune response, inflammation, vasculoprotective properties, and tissue remodeling. It is primarily produced by type-2 helper T (Th2) lymphocytes during inflammation, where it acts to suppress the production of several proinflammatory cytokines.^30^ IL-10 has gained significant attention due to its ability to suppress inflammatory and proliferative vasculopathy.^30^ IL-10 inhibits inflammation by decreasing the production of the inflammatory cytokines, thus exerting anti-inflammatory effects. Additionally, IL-10 activates signaling pathways that enhance the expression of anti-inflammatory genes.^31^

The pathological process of PH is characterized by abnormal serum levels of proinflammatory cytokines such as IL-1 and IL-6.^32^ Patients with PH often exhibit elevated baseline serum levels of IL-10, suggesting a protective anti-inflammatory response to the ongoing injury.^33^ Therefore, IL-10 has become a focal point in potential therapies aimed at combating fibrosis linked to inflammation.^34^

IL-10 plays an indirect yet crucial role in limiting cardiac injury and fibrosis. Its signaling pathways, particularly through STAT3, promote the recruitment and retaining bone marrow-derived endothelial progenitor cells at the site of heart injury, thereby influencing repair and regeneration.^35,36^ Much of the experimental data in the literature suggest a beneficial role for IL-10 in PH. For instance, in the MCT-PH rat model, intravenous administration of IL-10 via adenoviral vector significantly improved survival rates and reduced mean pulmonary artery pressures.^30^ Similarly, recombinant IL-10 administration improved ventricular function, reduced hypertrophic remodeling, attenuated cardiac fibrosis and proliferative vasculopathy, and lowered mortality rate.^37^

Systemic expression of IL-10 also enhanced survival in MCT-PH rats, prevented the development of right ventricular hypertrophy, medial hypertrophy of the pulmonary artery, reduced macrophage accumulation, vascular cell proliferation, and lowered pulmonary tissue levels of TGF-β1 and IL-6, which are critical in PH progression.^38^ Consistent with previous studies using MCT-PH rat model, increased IL-10 induced by specific administration of IL-10 significantly inhibited the expression of IL-1β, IL-6 and TNF-α in the right ventricle, and attenuated fibrosis in both the right and left ventricles.^30^ Overall, IL-10 modulates the dynamics of cytokine networks involved in PH-induced heart remodeling, potentially exerting its effects at various sites.

Our results evidenced that D-L augmented the expression of IL-10 in right ventricular tissue. IL-10 can up-regulate endogenous anti-cytokines and down-regulate pro-inflammatory cytokine receptors, and shows potent anti-inflammatory properties, repressing the expression of IL-1β, IL-6 and TNF-α by activated macrophages.^39^ A previous study has demonstrated that IL-10, delivered by an intramuscular injection of an adeno-associated virus vector, exerts multiple preventive effects on inflammatory and proliferative vascular remodeling in PH, such as reduced macrophage accumulation, vascular cell proliferation, and pulmonary tissue levels of TGF-1 and IL-6.^30^ Lastly, D-L was already described to modulate inflammatory response in the heart.^40^

In line with our result, in a previous study^12^ it was found that inflammation covered almost the entire course of PH and inflammatory mediator levels are increased during the progression from acute and chronic inflammatory phase.^12^ The IL-1β is one of the first cytokines to be elevated in the course of the experimental MCT model in rats, and it has been shown to be related to atrial and ventricular arrhythmias observed in a range of heart disease.^41,42^

D-L can ameliorate cardiac injury induced by CCl_4_ intoxication through its antioxidant and anti-inflammatory potential.^40^Moreover, D-L anti-inflammatory properties have previously been proven through the inhibition of redox-dependent NF-κB and other downstream inflammatory cytokines which are key players in exhibition of inflammation.^40^

In line with this concept, D-L was already described to modulate inflammatory response in the heart.^40^ In our study, we provide for the first time a more specific and detailed analysis showing that *in vivo* D-L administration is able to specifically modulate the inflammatory status of the right ventricle, which is severely affected in the MCT-PH- induced heart remodeling.

## Conclusion

Based on the findings, it can be concluded that *in vivo* administration of D-L can reduce the formation of tissue fibrosis in the MCT-PH model in young rats, as summarized in Central Figure. Moreover, D-L restores electrocardiographic changes and increase the expression of anti-inflammatory cytokines. Overall, D-L may be a promising anti-inflammatory and antioxidant agent against PH and cardiac dysfunction.
